# Defining cases of asthma, eczema and allergic rhinitis using electronic health records in the Born in Bradford birth cohort

**DOI:** 10.1111/cea.14291

**Published:** 2023-02-09

**Authors:** Sergio Souza da Cunha, Gillian Santorelli, Lucy Pembrey

**Affiliations:** ^1^ Bradford Teaching Hospitals NHS Trust Bradford Institute for Health Research Bradford UK; ^2^ London School of Hygiene and Tropical Medicine London UK

**Keywords:** allergic diseases, asthma, allergic rhinitis, children, eczema, electronic health records

## Abstract

**Key Messages**

• In electronic health records, the accuracy of diagnostic codes to define outcomes can be uncertain

• The accuracy can vary in different settings, doctors and practices, even with validated codes

• We recommend definitions combining codes previously described and other codes available in the records

To the Editor,

In studies based on electronic health record (EHR) databases, diagnostic codes are commonly used to define clinical outcomes. However, the accuracy of the codes depends on several factors, such as whether the medical diagnosis is correct and the opportunity for physical examination (ascertainment process), and validity can vary between datasets.^1^, ^2^ The diagnosis of asthma and allergic diseases (AAD) in young children is particularly challenging: the symptoms are intermittent and the differential diagnosis is difficult.^3^ Therefore, most diagnoses rely on response to treatment and parental report of symptoms that can be influenced by past experiences of diseases in the children and parents, which in turn can lead to recall bias. The impact of disease misclassification can be important depending on whether it is differential or non‐differential, and whether it is dependent on other errors.^4^

We recently analysed the association between exposure to antibiotics and the risk of AAD (asthma, atopic eczema, and allergic rhinitis^5^, ^6^), in children participating in the Born in Bradford (BiB) birth cohort study.^7^ Briefly, 12,453 pregnant women were recruited to BiB between 2007 and 2010, resulting in the births of over 13,500 children. Consent for health record linkage was obtained, and has been achieved for approximately 98% of participants. In total, 13,044 children were linked to EHR. The protocol for the antibiotics study, written before the study started, can be found in reference 5.^5^ In this letter, we present our approach to defining AAD outcomes using CTV3 Read codes (coded clinical terms designed for use in EHR in the NHS in the UK) and British National Formulary (BNF) codes for prescriptions of medications.

Initially, we planned to follow the common practice of using only validated definitions described in previous studies using EHR to ensure comparability. However, we reflected over some issues: diagnostic procedures are not standardised; the codes used and their frequency can vary across different settings and doctors; and there could be cases that are not recorded with the validated codes. Conversely, including all Read codes found in our EHR relating to our outcomes could lead to bias where Read codes are used for non‐cases (e.g., family history of asthma).

Using some of the methods recommended for developing clinical codelists,^8^ we first conceived conceptual definitions for each disease based on available data. Then, we searched for diagnoses in our EHR database in two ways: (1) using diagnostic codes described in previous studies, and (2) using case‐insensitive text mining of the term definitions that accompany Read codes that could indicate diagnosis of AAD. For asthma, we found a large number of terms that required us to adopt a pragmatic approach to short listing. The authors SSC and LP selected all codes describing diagnoses, current adherence to treatment and control assessments, and excluded those describing asthma screening or which were considered too vague. For atopic eczema and hay fever, all codes found were related to the diagnosis and did not require the steps we employed for asthma. Additionally, we searched for BNF codes for the most common medications used to treat AAD (including generic and brand names). We discussed our definitions and lists of Read/BNF codes with clinicians and other researchers with expertise in AAD and agreed on the final definitions.

To deal with levels of uncertainty of whether or not the presence of a Read code for AAD reflected a confirmed diagnosis of AAD, we created two definitions for each outcome. The first definition was regarded as being more specific compared to the second for asthma and atopic eczema. The final case definitions are detailed in Table 1, and the CTV3 Read codes can be found at https://doi.org/10.17037/DATA.00003098.

**TABLE 1:** Case definitions for asthma, atopic eczema and allergic rhinitis

Outcome definitions	Description
Asthma	
1st definition	A child with at least one selected Read code for asthma at age 6 years or older or at age 3‐5 years AND at least 3 prescriptions per year at age 6 years or older (for at least one year) of bronchodilators or inhaled corticosteroids or leukotriene receptor antagonists
2nd definition	A child with at least one selected Read code for asthma at age 3 years or older, irrespective of prescription of medication
Atopic eczema	
1st definition	A child with at least one selected Read code for eczema from age 1 year onwards AND at least two prescriptions of eczema‐related treatment within 90 days before or 365 days after the first recorded eczema diagnosis defined by the presence of a Read code
2nd definition	A child with at least one selected Read code for eczema from age 1 year onwards, irrespective of prescriptions
Allergic rhinitis	
1st definition	A child with at least one selected Read code for allergic rhinitis or allergic conjunctivitis at any age
2nd definition	A child with at least one selected Read code for allergic rhinitis or allergic conjunctivitis with at least one Read code during hay fever season (1st March to 31st July) at any age AND at least one prescription during at least one hay fever season of: anti‐histamines or intra‐nasal corticosteroids

For asthma, differential diagnosis can be challenging in children under 5 years of age. We therefore based our first definition on (1) those with selected Read codes at 6 years old irrespective of prescriptions, or (2) those with Read codes only between 3 and 5 years but with regular prescriptions for asthma at ≥ 6 years of age. This demonstrates repeated prescriptions when the diagnosis is made with more certainty. The second definition was defined by the presence of the selected Read codes at age 3 years or older, irrespective of a prescription being issued. For atopic eczema, we selected CTV3 Read codes adapted from previous studies.^9^ We excluded infants <1 year old as children of this age frequently have rashes which can be misdiagnosed as eczema. The first definition for eczema included the presence of selected Read codes plus at least two prescriptions of eczema‐related treatment within 90 days before or 365 days after the first recorded eczema diagnosis defined by the presence of a Read code. For the second definition we considered only the presence of relevant Read codes. Due to the wide availability of remedies available over the counter (OTC), our first definition for allergic rhinitis was based on the presence of Read codes only. The second definition, regarded as more specific, was restricted to a subset of children for whom a Read code was present between 1 March and 31 July and who were also prescribed medication. This period corresponds to the season of allergic rhinitis induced by pollen allergy (hay fever).

We compared the different prescriptions used in our case definitions against the Read codes as the reference standard, using positive and negative predictive values (PPV and NPV, respectively).^2^ PPV represents the proportion of children who had a Read code out of those who were prescribed medication; NPV refers to the proportion who did not have a Read code out of those who were not prescribed medication. For asthma, where all medications are dispensed on prescription, the PPV was high for inhaled corticosteroids and leukotriene receptor antagonists. The NPV was high for all three medications, with bronchodilators and inhaled corticosteroids being prescribed for most cases (Table 2). This suggests that prescriptions as additional criteria can be of particular relevance for asthma. All medications are on prescription, so the prescriptions are accurately recorded. Children with early wheeze which resolves before 6 years old can be excluded. For eczema, the majority of prescriptions issued were for topical corticosteroids and emollients, both of which had a low PPV but high NPV. For allergic rhinitis, medications were often prescribed in the absence of Read codes, suggesting that prescriptions are less useful to differentiate cases from non‐cases. Thus, for eczema and allergic rhinitis where remedies are widely available OTC (with the exception of topical calcineurin inhibitors for eczema), a relevant prescription being issued did not necessarily reflect the diagnosis of disease. Given that recurrent upper respiratory tract infections (URTI) are common in young children, it is possible that some cases of allergic rhinitis based only on Read codes may be URTI misclassified as allergic rhinitis.

**TABLE 2:** Number of children according to disease status based only on Read codes, and number of children with prescriptions of medications, among 13,044 children with linked electronic health records

Medication and availability^a^	Medication prescribed	Relevant Read code recorded N (%)		PPV^b^ (95% CI)	NPV^c^ (95% CI)
		Yes	No		
**Asthma** ^d^		1,303	11,741		
Bronchodilators (Rx)	Yes	883 (67.8)	556 (4.7)	0.61 (0.59, 0.64)	0.96 (0.96, 0.97)
	No	420 (32.2)	11,185 (95.3)		
Inhaled corticosteroids (Rx)	Yes	637 (48.9)	66 (0.6)	0.91 (0.88, 0.93)	0.95 (0.94, 0.95)
	No	666 (51.1)	11,675 (99.4)		
Leukotriene receptor antagonists (Rx)	Yes	186 (14.3)	18 (0.2)	0.91 (0.87, 0.95)	0.91 (0.91, 0.92)
	No	1,117 (85.7)	11,723 (99.8)		
Any of the above medications (Rx)	Yes	930 (71.4)	564 (4.8)	0.62 (0.60, 0.65)	0.97 (0.96, 0.97)
	No	373 (28.6)	11,177 (95.2)		
**Eczema** ^e^		3,420	9,624		
Topical corticosteroid (OTC/Rx)	Yes	3,196 (93.5)	4,902 (50.9)	0.39 (0.38, 0.41)	0.95 (0.95, 0.96)
	No	224 (6.5)	4,722 (49.1)		
Emollients (OTC/Rx)	Yes	3,420 (100.0)	9,564 (99.4)	0.26 (0.26, 0.27)	1.00 (0.94, 1.00)
	No	0 (0)	60 (0.6)		
Topical calcineurin inhibitors (Rx)	Yes	59 (1.7)	23 (0.2)	0.72 (0.62, 0.82)	0.74 (0.73, 0.75)
	No	3,361 (98.3)	9,601 (99.8)		
Systemic corticosteroids (Rx)	Yes	606 (17.7)	1,102 (11.5)	0.35 (0.33, 0.38)	0.75 (0.74, 0.76)
	No	2,814 (82.3)	8,522 (88.5)		
Any of the above medications (OTC/Rx)	Yes	3,420 (100)	9,564 (99.4)	0.26 (0.26, 0.27)	1.00 (0.94, 1.00)
	No	0 (0)	60 (0.6)		
**Allergic rhinitis** ^f^		985	12,059		
Antihistamines (OTC/Rx)	Yes	825 (83.8)	3,828 (31.7)	0.18 (0.17, 0.19)	0.98 (0.98, 0.98)
	No	160 (16.2)	8,231 (68.3)		
Intranasal corticosteroids (OTC/Rx)	Yes	430 (43.7)	2,815 (23.3)	0.13 (0.12, 0.14)	0.94 (0.94, 0.95)
	No	555 (56.3)	9,244 (76.7)		
Any of the above medications (OTC/Rx)	Yes	879 (89.2)	5,481 (45.5)	0.14 (0.13, 0.15)	0.98 (0.98, 0.99)
	No	106 (10.8)	6,578 (54.5)		

^**a**^ If the medication is dispensed only on prescription (Rx) or over‐the‐counter (OTC).

^**b**^ Positive predictive value: the proportion of children who had a relevant Read code among those who were issued a prescription.

^c^ Negative predictive value: the proportion of children who did not have a relevant Read code among those who were not issued a prescription.

^**d**^ For asthma, ≥3 prescriptions at 6 years old.

^**e**^ For eczema, ≥2 prescriptions at any age between 0 to 7 years old; immunoregulators are not presented in the table because there were only 8 children with prescriptions.

^f^ For allergic rhinitis, prescriptions at any age between 0 to 6 years old during hay fever season.

In conclusion, we have presented case definitions for asthma, atopic eczema and allergic rhinitis using UK EHR data on GP‐recorded diagnoses and prescriptions. We combined definitions adapted from previous studies with text mining of Read codes and amended our definitions in consultation with experts in this field. We recommend that researchers consider using this approach in similar UK studies to deal with uncertainties in the case definitions.

## AUTHOR CONTRIBUTIONS

All authors designed the study. Sergio Souza da Cunha searched the databases, conducted the analyses and drafted the manuscript. Gillian Santorelli and Lucy Pembrey revised the manuscript. All authors approved the final version of the manuscript.

## CONFLICT OF INTEREST STATEMENT

None of the authors have conflicts of interest to declare.

## FUNDING INFORMATION

BiB receives core infrastructure funding from the Wellcome Trust (WT101597MA) and a joint grant from the UK Medical Research Council (MRC) and Economic and Social Science Research Council (ESRC) (MR/N024397/1). This study receives funding from the National Institute for Health and Care Research, UK (HTA Project: 16/150/06).

## ETHICS STATEMENT

The Born in Bradford cohort study has Bradford Research Ethics Committee approval (Ref: 07/H1302/112). The NIHR antibiotics study has Health Research Authority (HRA) and Health and Care Research Wales (HCRW) approval (ref: 238908).

## Data Availability

The data that support the findings of this study are openly available in LSHTM Data Compass at https://doi.org/10.17037/DATA.00003098.
